# Moisture-Assisted near-UV Emission Enhancement of Lead-Free Cs_4_CuIn_2_Cl_12_ Double Perovskite Nanocrystals

**DOI:** 10.1021/acs.nanolett.1c03822

**Published:** 2021-12-23

**Authors:** Maning Liu, Sri Kasi Matta, Harri Ali-Löytty, Anastasia Matuhina, G. Krishnamurthy Grandhi, Kimmo Lahtonen, Salvy P. Russo, Paola Vivo

**Affiliations:** ‡ Hybrid Solar Cells, Faculty of Engineering and Natural Sciences, Tampere University, P.O. Box 541, Tampere FI-33014, Finland; § Australian Research Council Centre of Excellence in Exciton Science, School of Science, RMIT University, Melbourne, Victoria 3000, Australia; ⊥ Surface Science Group, Photonics Laboratory, Tampere University, P.O. Box 692, Tampere FI-33014, Finland; ∥ Faculty of Engineering and Natural Sciences, Tampere University, P.O. Box 692, Tampere FI-33014, Finland

**Keywords:** lead-free double perovskite nanocrystals, moisture-assisted, near-UV emission, 2D nanoplatelets, self-trapped excitons

## Abstract

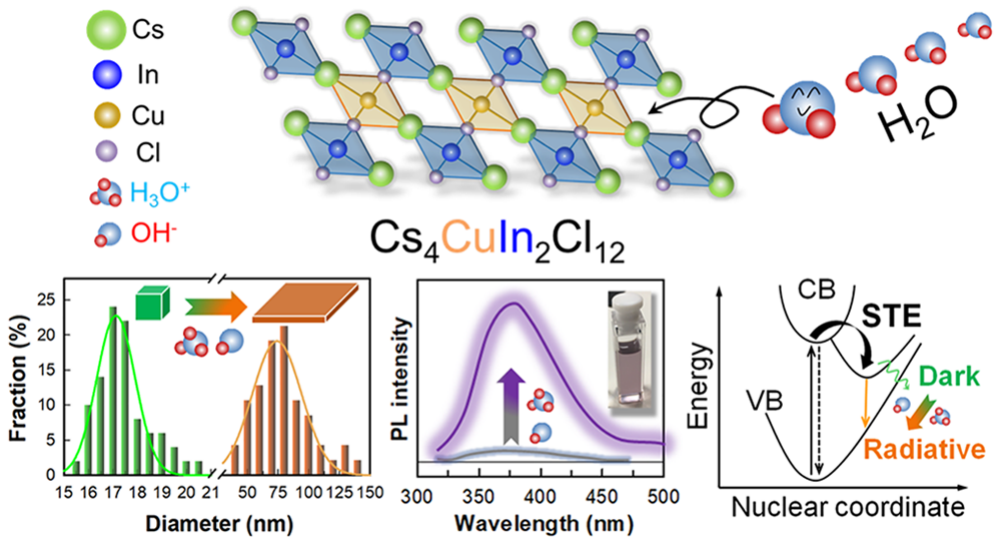

Lead-based halide perovskite nanocrystals (NCs) are recognized as emerging emissive materials with superior photoluminescence (PL) properties. However, the toxicity of lead and the swift chemical decomposition under atmospheric moisture severely hinder their commercialization process. Herein, we report the first colloidal synthesis of lead-free Cs_4_CuIn_2_Cl_12_ layered double perovskite NCs via a facile moisture-assisted hot-injection method stemming from relatively nontoxic precursors. Although moisture is typically detrimental to NC synthesis, we demonstrate that the presence of water molecules in Cs_4_CuIn_2_Cl_12_ synthesis enhances the PL quantum yield (mainly in the near-UV range), induces a morphological transformation from 3D nanocubes to 2D nanoplatelets, and converts the dark transitions to radiative transitions for the observed self-trapped exciton relaxation. This work paves the way for further studies on the moisture-assisted synthesis of novel lead-free halide perovskite NCs for a wide range of applications.

Benefiting from their reduced toxicity, compositional tunability, and superior structural stability,^1−4^ lead-free double perovskites have attracted increasing attention in recent years both in the form of bulk films^5−8^ and colloidal nanocrystals (NCs),^9−12^ as substitutive materials of lead-based halide perovskites for potential commercial applications, i.e., light-emitting diodes (LEDs),^13^ solar cells,^14^ and photodetectors.^15^ Compared to the well-investigated cubic double perovskites with structure Cs_2_M(I)M(III)X_6_ (M(I), Ag^+^, Cu^+^, etc.; M(III), Bi^3+^, Sb^3+^, In^3+^, etc.; X, Cl^–^, Br^–^, I^–^),^16−20^ the recently discovered vacancy-ordered layered double perovskites with formula Cs_4_M(II)M(III)_2_X_12_ (M(II), Cu^2+^, Mn^2+^, etc.) have demonstrated several attractive figures of merit including large compositional space, direct bandgap nature, and outstanding structural stability.^21−24^ The layered double perovskite structure comprises one layer of [M(II)X_6_]^4–^ octahedra inserted in between two layers of [M(III)X_6_]^3–^ octahedra. As a representative, Cs_4_CuSb_2_Cl_12_ has been successfully synthesized both in the form of a single-crystalline powder^22^ and NCs,^23,25^ exhibiting a narrow direct bandgap (1.0–1.8 eV) and impressive stability, which still suffers from the high toxicity of the Sb element and the absence of emission at room temperature. To overcome these drawbacks, Cs_4_CuIn_2_Cl_12_, with toxic Sb^3+^ substituted by relatively nontoxic In^3+^, could be a promising candidate to fulfill the requirement of compositional engineering and optical tunability particularly in the UV range. Yet, to date, the synthesis of Cs_4_CuIn_2_Cl_12_ layered double perovskites has not been reported for either bulk film or NCs.

Herein, we report the first-ever colloidal synthesis of lead-free Cs_4_CuIn_2_Cl_12_ layered double perovskite NCs using a modified hot-injection method.^20,25^ The synthetic details are described in the Supporting Information. Although a standard hot-injection reaction in a moisture-free environment resulted in Cs_4_CuIn_2_Cl_12_ with a very low photoluminescence quantum yield (PLQY) of 0.12%, we found that the handling of synthesis precursors in the presence of moisture (RH ∼ 40%) enhances the PLQY by more than 1 order of magnitude up to 1.70%. Water-assisted in situ synthesis has been recognized as an effective strategy to tune the optical properties and stability for both lead-based^26,27^ and lead-free halide perovskite NCs^28,29^ via the controlling of NCs size, shape, and crystallinity. Nevertheless, there is still a lack of deep understanding of how water influences the NC growth regime and corresponding PL property, especially for lead-free double perovskite NCs. The introduction of moisture in the precursor (namely, “wet” precursor conditions) of Cs_4_CuIn_2_Cl_12_ NCs (w-Cs_4_CuIn_2_Cl_12_) induces the morphological transformation from 3D nanocubes (NCus) to 2D nanoplatelets (NPLs), driven by the ionized H_3_O^+^ and OH^–^ from the water content as additional capping ligands. The ultrafast transient absorption studies suggest a strengthened self-trapped exciton (STE) effect for w-Cs_4_CuIn_2_Cl_12_ NCs compared to the NCs synthesized in “dry” conditions (d-Cs_4_CuIn_2_Cl_12_ NCs), resulting in the conversion of dark transitions into radiative transitions, which directly contributes to the PLQY.

The absorption and PL spectra of as-synthesized d-Cs_4_CuIn_2_Cl_12_ and w-Cs_4_CuIn_2_Cl_12_ NC suspensions are presented in panels a and b of Figure 1. We conducted Tauc analysis for both direct (Figure 1a, inset) and indirect (Figure S1) transitions, with distinguishable direct bandgap energies (3.82 eV for d-Cs_4_CuIn_2_Cl_12_ and 3.56 eV for w-Cs_4_CuIn_2_Cl_12_), which are consistent with the observed direct bandgap nature of other In-based double perovskites.^30^ However, we note that the bandgap of the Cs_4_CuIn_2_Cl_12_ NCs (3.56 or 3.82 eV) in this work is remarkably larger than the one reported for the Cs_4_CuSb_2_Cl_12_ double perovskite analogue (1.0–1.8 eV).^22,25^ We attributed the bandgap difference mainly to the copper-induced additional near valence band maximum (VBM) states along with the chlorine,^31^ i.e., intermittent states that are discontinuous in the case of Cs_4_CuIn_2_Cl_12_ based on our DFT calculation results for density of states (DOS) (see Figure S2). On the other hand, Cs_4_CuSb_2_Cl_12_ double perovskites possess a continuous band, suggesting that the extension up to Fermi energy level (set as zero) can create a net large bandgap in the Cs_4_CuIn_2_Cl_12_ double perovskites. However, in both cases, Cl 3p and Cu 3d orbitals dominate the valence band (VB) energy levels. The absorption spectrum of d-Cs_4_CuIn_2_Cl_12_ NCs peaks at 269 nm, and their weak emission maximum is at 374 nm. Upon the involvement of moisture in the reaction, the absorption spectrum of w-Cs_4_CuIn_2_Cl_12_ NCs exhibits a slight red-shift, which has also been observed for the formation of CsPbBr_3_ NPLs upon the addition of water molecules.^26^ A long absorption tail in the visible range is observed in the absorption spectrum of w-Cs_4_CuIn_2_Cl_12_ NCs, which contributes to the visualization of a violet color in suspension compared to the whitish color observed for d-Cs_4_CuIn_2_Cl_12_ NCs (Figure 1b, inset). It is also noteworthy that the absorption coefficient of w-Cs_4_CuIn_2_Cl_12_ NCs is clearly higher than that of d-Cs_4_CuIn_2_Cl_12_ NCs particularly in the range of 300–700 nm. The absorption coefficients of halide perovskite materials are mainly determined by two factors including the excitonic contribution and continuum states.^32^ We thus speculate that the continuum states are similar between d-Cs_4_CuIn_2_Cl_12_ and w-Cs_4_CuIn_2_Cl_12_ NCs based on our DFT simulation for DOS; however, the involvement of water molecules could effectively enhance the excitonic contribution in the case of w-Cs_4_CuIn_2_Cl_12_ NCs as evident by the observed strengthened STE effect that will be discussed later on, eventually resulting in the higher absorption coefficient for w-Cs_4_CuIn_2_Cl_12_ NCs compared to the case of d-Cs_4_CuIn_2_Cl_12_ NCs. Moreover, the corresponding PL spectrum of w-Cs_4_CuIn_2_Cl_12_ NCs experiences a significant amplitude enhancement mainly in the near-UV range (320–400 nm) by showing an absolute PLQY of 1.70%, which is more than 1 order of magnitude higher than that (0.12%) of d-Cs_4_CuIn_2_Cl_12_ NCs. A relatively broad (fwhm = 73 nm) and asymmetric PL spectral shape, with a long emission tail beyond 550 nm, is detected in the case of w-Cs_4_CuIn_2_Cl_12_ NCs. The enhanced PL intensity of w-Cs_4_CuIn_2_Cl_12_ NCs could be attributed to the STEs originating from the water-induced Jahn–Teller distortion of the [M(II)Cl_6_] (i.e., [CuCl_6_]) octahedra in the excited state, as has also been observed for other types of lead-free perovskites, such as Cs_2_AgInCl_6_^33^ and Rb_2_InCl_5_.^28^ It is worth mentioning that the Stokes shift (∼108 nm) for the w-Cs_4_CuIn_2_Cl_12_ NCs in this work is smaller than other reported shifts (>150 nm) for STE lead-free double perovskites,^33^ which could be related to the different degrees of Jahn–Teller distortion of the [CuCl_6_] octahedra induced by the diverse water amounts. It has been reported that the greater degree of Jahn–Teller distortion, the more red-shifted the STE emission.^28^ Because the water content in the NC reaction comes only from the atmospheric moisture (RH ∼ 40%), this may suggest that the degree of Jahn–Teller distortion, and thus the Stokes shift, can be further increased by introducing more water molecules, which will be investigated separately. In addition, the PL excitation (PLE) spectrum (Figure 1b) of w-Cs_4_CuIn_2_Cl_12_ NCs, monitored at the emission peak (381 nm), presents the multiple peaks that have also been observed for other types of In-based double perovskites such as Cs_2_AgInCl_6_, which are related to different defect states and/or surface-related states.^33^ We thus assign the first PLE peak (∼305 nm) to the direct transition close to the band edge, while attributing the second (∼325 nm) and third peaks (∼343 nm) to the two different STE states, i.e., surface localized carriers and Jahn–Teller distortion of the [CuCl_6_] octahedra in the excited state, respectively. Interestingly, only one peak (∼301 nm) is observed in the PLE spectrum of d-Cs_4_CuIn_2_Cl_12_ NCs, in agreement with the negligible STE state in the case of d-Cs_4_CuIn_2_Cl_12_ because of the absence of the water molecules in the reaction, resulting in a weak emission signal. The optical properties of as-synthesized Cs_4_CuIn_2_Cl_12_ NCs are summarized in Table 1.

**Figure 1:**
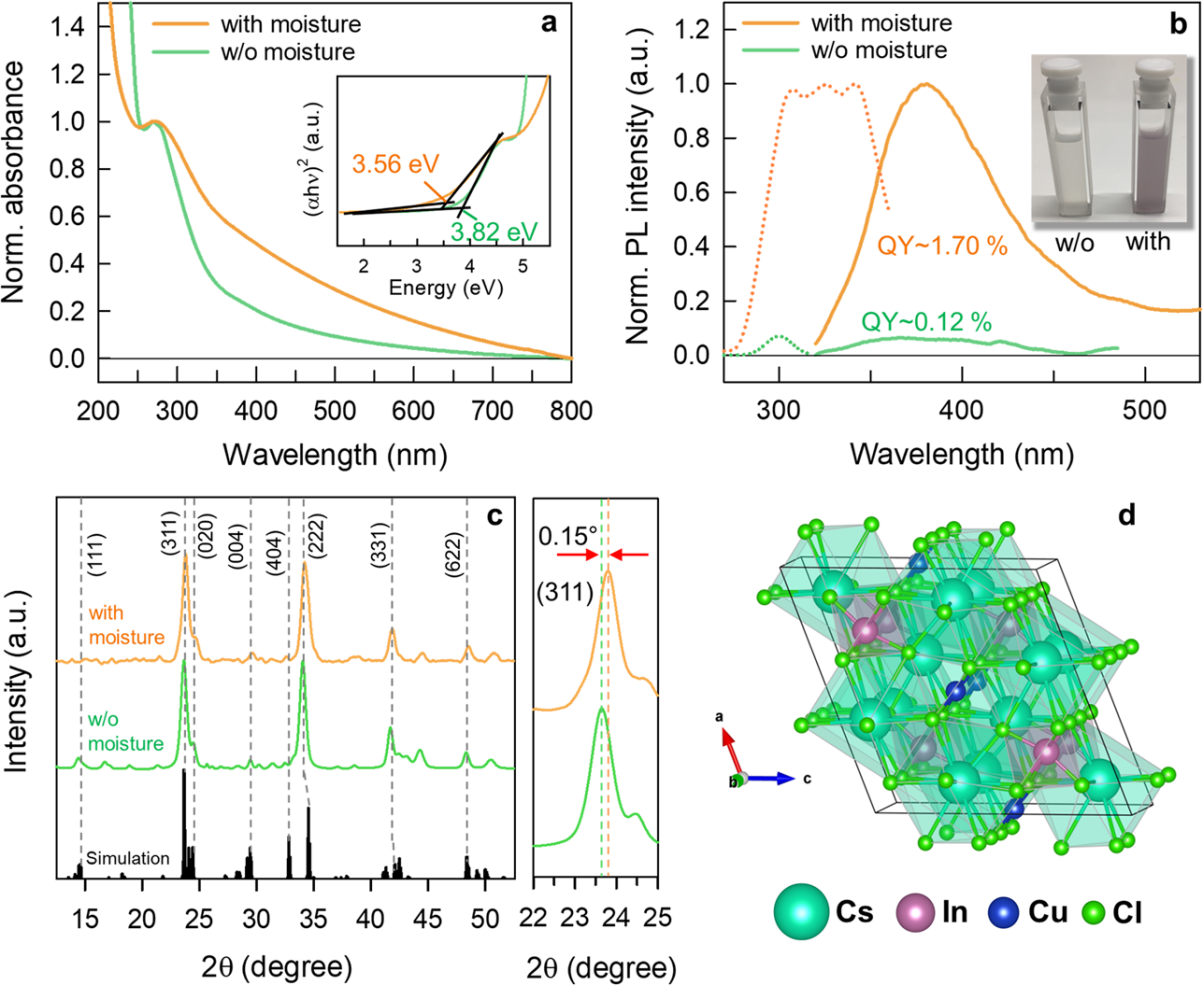
(a) Absorption spectra and (b) photoluminescence (PL, excitation at 300 nm) spectra of Cs_4_CuIn_2_Cl_12_ NC suspensions synthesized with (w-Cs_4_CuIn_2_Cl_12_) and without (w/o) (d-Cs_4_CuIn_2_Cl_12_) moisture, combined with a PL excitation (PLE, emission at 381 nm) spectra (dotted lines) of w- and d-Cs_4_CuIn_2_Cl_12_ NCs. Insets: (a) Tauc plots and (b) a photograph of the NC toluene suspensions. (c) X-ray diffraction (XRD) patterns of d-Cs_4_CuIn_2_Cl_12_ and w-Cs_4_CuIn_2_Cl_12_ NCs, comparied with the simulated XRD pattern for bulk Cs_4_CuIn_2_Cl_12_ in a pure phase. (d) Crystal structure of a Cs_4_CuIn_2_Cl_12_ unit cell.

**Table 1:** Optical Properties of As-Synthesized Cs_4_CuIn_2_Cl_12_ NCs

NCs	aλ_abs_ (nm)	bλ_PL_ (nm)	cfwhm (nm)	PLQY (%)	d*E*_g_ (eV)
d-Cs_4_CuIn_2_Cl_12_	269	374	95	0.12	3.82
w-Cs_4_CuIn_2_Cl_12_	273	381	73	1.70	3.56

a First exciton peak.

b Emission peak.

c Full width at half-maximum.

d Bandgap.

The X-ray diffraction (XRD) patterns of Cs_4_CuIn_2_Cl_12_ NCs formed in the presence or absence of moisture in the reaction show a negligible deviation from the simulated pattern of bulk Cs_4_CuIn_2_Cl_12_ in a pure phase without considering any impurities (Figure 1c, left), determining a monoclinic crystal structure with a space group of C2/*m*. The set of characteristic peaks positioned at 23.6, 24.5, and 34.0° were assigned to (311), (020), and (222) planes, corresponding to the Cs_4_CuIn_2_Cl_12_ layered double perovskite crystal phase with distorted [CuCl_6_] octahedra, which has also been observed for Cs_4_CuSb_2_Cl_12_ NCs.^25^ On the basis of our simulation results (Table S1), the lattice parameters were calculated as *a* = 13.18 Å, *b* = 7.29 Å, *c* = 13.16 Å, and β = 111.8° with a unit cell volume of 1174.6 Å^3^ (see the crystal structure of one Cs_4_CuIn_2_Cl_12_ unit cell in Figure 1d). It is noteworthy that the XRD pattern of w-Cs_4_CuIn_2_Cl_12_ NCs shows a slightly upward shift in the peak positions compared to that of the reference d-Cs_4_CuIn_2_Cl_12_ NCs, e.g., from 23.66 to 23.81° at (311) (Figure 1c, right), indicating that the water molecules indeed trigger the shrinkage of the unit cell during the NC growth. The stabilities of the NCs were further studied by measuring XRD patterns as a function of storage time. Specifically, we studied the intensity evolution of the characteristic peak at the (311) plane for two NC samples (Figure S3). Up to 4 weeks storage, w-Cs_4_CuIn_2_Cl_12_ NCs retain 95% of the initial intensity, whereas a more than 50% drop in intensity occurs for d-Cs_4_CuIn_2_Cl_12_ NCs, indicating that the moisture-assisted NCs possess good structural stability under ambient conditions (RH ∼ 40%). Energy-dispersive X-ray spectroscopy (EDS) analysis elucidated the actual elemental ratios of Cs:Cu:In:Cl as 4.0:0.7:1.9:12.0 and 4.0:0.8:1.9:11.9 for the d-Cs_4_CuIn_2_Cl_12_ and w-Cs_4_CuIn_2_Cl_12_ NCs, respectively, both matching well the nominal stoichiometric ratio of Cs_4_CuIn_2_Cl_12_. The EDS layered images of w-Cs_4_CuIn_2_Cl_12_ NCs film (Figure S4) for different compositional elements show the relatively homogeneous elemental distribution on the surface of the NC film with a sign of Cu segregation toward the grain boundary that could be induced by the X-ray beam during the measurement, which has also been observed for other Cu-based compounds.^34,35^ Our inductively coupled plasma mass spectroscopy (ICP-MS) and X-ray photoelectron spectroscopy (XPS) analyses (Tables S2 and S3) further confirmed the ion ratios of Cs:Cu:In and Cu:In:Cl matching the desired stoichiometric ratios (4:1:2 and 1:2:12) for both NCs samples, respectively. The concentration of Cs detected from the XPS analysis should be considered as an underestimate due to the X-ray and electron-beam-induced depletion of Cs during the measurements.^36^

The surface composition of w-Cs_4_CuIn_2_Cl_12_ and d-Cs_4_CuIn_2_Cl_12_ NC samples in film were further analyzed by XPS. The chemical compositions of surfaces are similar except for the difference observed in the O 1s XPS spectrum in Figure S5. The surface of the d-Cs_4_CuIn_2_Cl_12_ NCs film is virtually free from oxygen, whereas an O 1s peak at 532.0 eV was measured for the w-Cs_4_CuIn_2_Cl_12_ NC film. This binding energy is higher than the ones of Cs, Cu, or In oxides^37^ but in the range of surface oxygen or peroxide species^38^ and water-related species, e.g., hydroxides and adsorbed or lattice hydroxyls.^39^ The result suggests the incorporation of water-mediated species within the w-Cs_4_CuIn_2_Cl_12_ NCs during the synthesis. More interestingly, the relative amount of oxygen species in w-Cs_4_CuIn_2_Cl_12_ is comparable to the amount of Cu (O/Cu = 0.8, see Table S2), which provides further support to the water-mediated lattice oxygen species. Only one chemical state was resolved for both Cs_4_CuIn_2_Cl_12_ samples for Cs (Cs 3d_5/2_ at 724.2 eV), Cu (Cu 2p_3/2_ at 931.7 eV), In (In 3d_5/2_ at 445.0–445.8 eV), and Cl (Cl 2p at 198.8–199.5 eV), which can be attributed to Cs^+^, Cu^+^, In^3+^, and Cl^–^, respectively (Figure S5).^37^ Except for Cu, the analyzed valence states are in accordance with the Cs^+^_4_Cu^2+^In^3+^_2_Cl^–^_12_ layered double perovskite structure. Cu 2p spectra presented in Figure S5b do not show the shakeup satellite features at 942 and 962 eV that are characteristic of Cu^2+^ compounds.^40^ A feasible explanation for the absence of expected Cu^2+^ from the surface is the photoreduction of Cu^2+^ to Cu^+^ that could had been induced by X-rays during the measurement.^41^ Indeed, Cai et al. measured a similar Cu 2p XPS spectrum showing no Cu^2+^ shakeup satellites for their Cs_4_CuSb_2_Cl_12_ NCs, whereas their electron paramagnetic resonance (EPR) measurements supported the presence of Cu^2+^.^25^

Transmission electron microscopy (TEM) measurements demonstrate that the d-Cs_4_CuIn_2_Cl_12_ NCs have a cubelike shape (Figure 2a) with an average diameter of 17.1 ± 1.6 nm (Figure 2b). High-resolution TEM (HRTEM) image (Figure 2c) highlights that d-Cs_4_CuIn_2_Cl_12_ NCs possess a well-defined crystalline structure with a lattice *d*-spacing of 0.376 nm, which is assigned to the (311) crystal plane, highly consistent with the crystalline direction determined from the selected area electron diffraction (SAED) pattern (Figure 2d). These, together with the previous XRD data (Figure 1c), further verified a monoclinic crystal structure. Interestingly, the NCs synthesized with the moisture in the precursor comprise the major population of 2D NPLs with an average lateral size of 74.1 ± 16.5 nm (Figure 2e) and an average thickness of ∼2.1 nm for one NPL, as estimated from the TEM image of the stacked NPLs (Figure 2e, inset). To further confirm the presence of stacked 2D NPLs, we analyzed the lower angles (i.e., 10–15°) in the XRD patterns of two NC samples (see Figure S6). One so-called basal low-angle (particularly for angles less than 14°) Bragg reflection (0*k*0)^42^ at (010) is observed for both d-Cs_4_CuIn_2_Cl_12_ and w-Cs_4_CuIn_2_Cl_12_ NCs. The Bragg angle at (010) shifts toward lower angle from 12.1° (for d-Cs_4_CuIn_2_Cl_12_) to 11.7° (for w-Cs_4_CuIn_2_Cl_12_), suggesting the presence of stacked w-Cs_4_CuIn_2_Cl_12_ NPLs, which has also been observed for CsPbBr_3_ NPLs.^43^ From the HRTEM image of NPLs (Figure 2g), the measured lattice *d*-spacing of 0.372 nm, also corresponding to (311) crystal facets (Figure 2h), was slightly lower than that (0.376 nm) of d-Cs_4_CuIn_2_Cl_12_ NCus, which is consistent with the shift in the peak positions of XRD data toward larger Bragg angles (Figure 1c). It is known that H_2_O molecules can be partly ionized into H_3_O^+^ and OH^–^ with the assistance of OA^–^ (OA, oleic acid) and OAm^+^ (OAm, oleylamine),^44^ which could act as additional capping ligands to activate the NC surface. We, thus, propose an NC growth scheme (Scheme 1) for better understanding the water-induced morphological transformation from 3D NCus to 2D NPLs for Cs_4_CuIn_2_Cl_12_ NCs. Upon the injection of TMS-Cl into the metal carboxylate precursors, there is a dynamic equilibrium between the attachment and detachment of capping ligands (i.e., OA and OAm) to influence the monomer attachment on the surface of the initially formed cluster.^45^ With a standard concentration of ligands (no moisture involved), there are many detachments of ligands from the cluster surface, and then with free ions (e.g., Cs^+^ and Cl^–^), more monomer attachments occur to further form nanocubes. When the water molecules are involved in the precursor, the ionized H_3_O^+^ and OH^–^ act as additional capping ligands, thus binding to the cluster surface and effectively blocking potential sites for monomer attachment, resulting in very few ligand detachments. Consequently, the NCs grow in the 2D direction to form nanoplatelets. This moisture-assisted shape change has been previously observed for CsPbBr_3_ NCs.^26^ In addition, the morphological transformation from 3D d-Cs_4_CuIn_2_Cl_12_ NCus to 2D w-Cs_4_CuIn_2_Cl_12_ NPLs is irreversible because the 2D NPLs still dominate the product population after 6 days of storage in a nitrogen-filled glovebox (see the TEM image comparison of 2D w-Cs_4_CuIn_2_Cl_12_ NPLs in the fresh and aged states in Figure S7).

**Figure 2:**
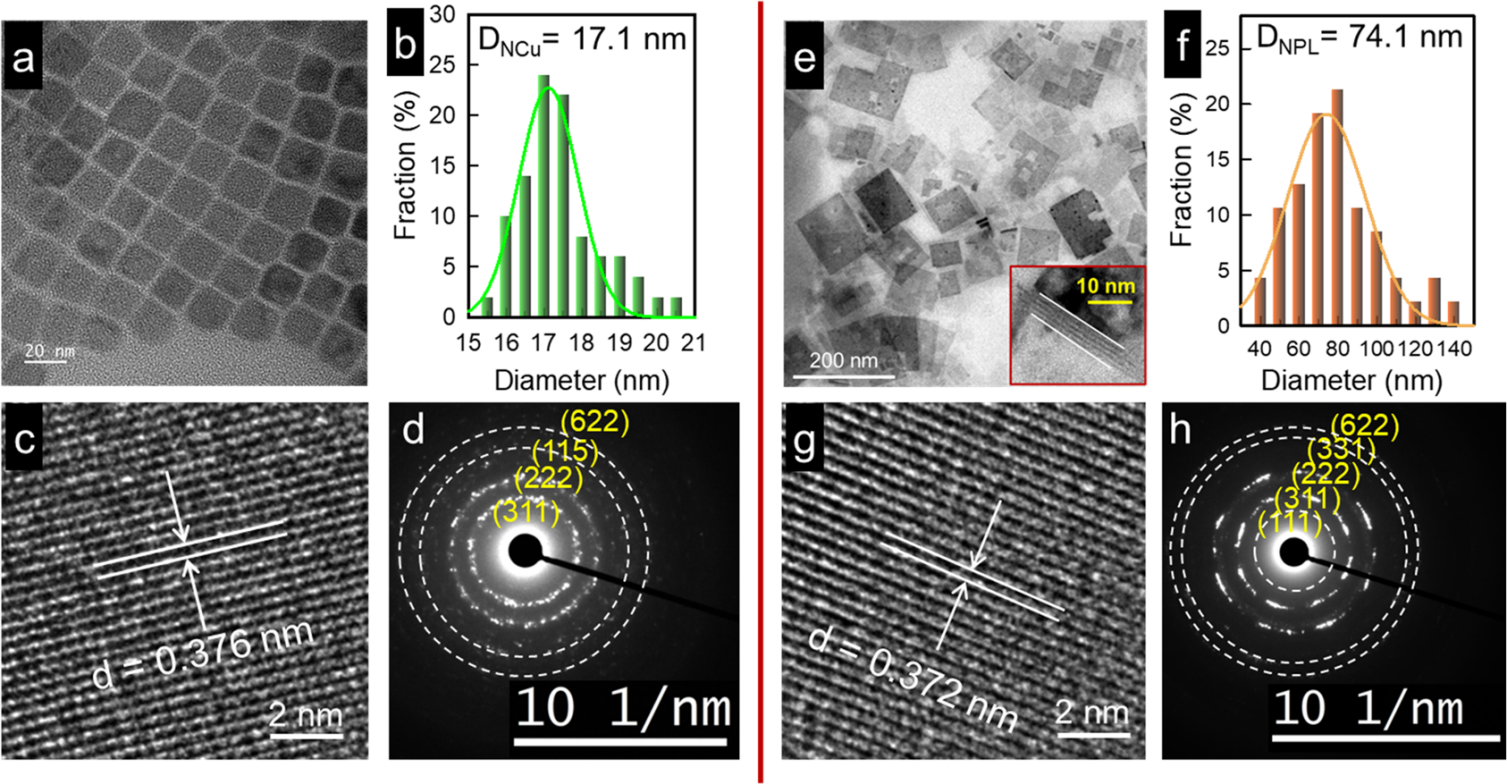
(a) Transmission electron microscopy (TEM) image and (b) size distribution histogram of d-Cs_4_CuIn_2_Cl_12_ NCs. (c) High-resolution TEM (HRTEM) image of a single d-Cs_4_CuIn_2_Cl_12_ NCu. (d) Selected area electron diffraction (SAED) pattern for the TEM image of NCus. (e) TEM image of w-Cs_4_CuIn_2_Cl_12_ NCs (the inset highlights the NPL stacking). (f) Size distribution histogram of w-Cs_4_CuIn_2_Cl_12_ NPLs. (g) HRTEM image of a single w-Cs_4_CuIn_2_Cl_12_ NPL. (h) SAED pattern for the TEM image of NPLs.

**Scheme 1:**
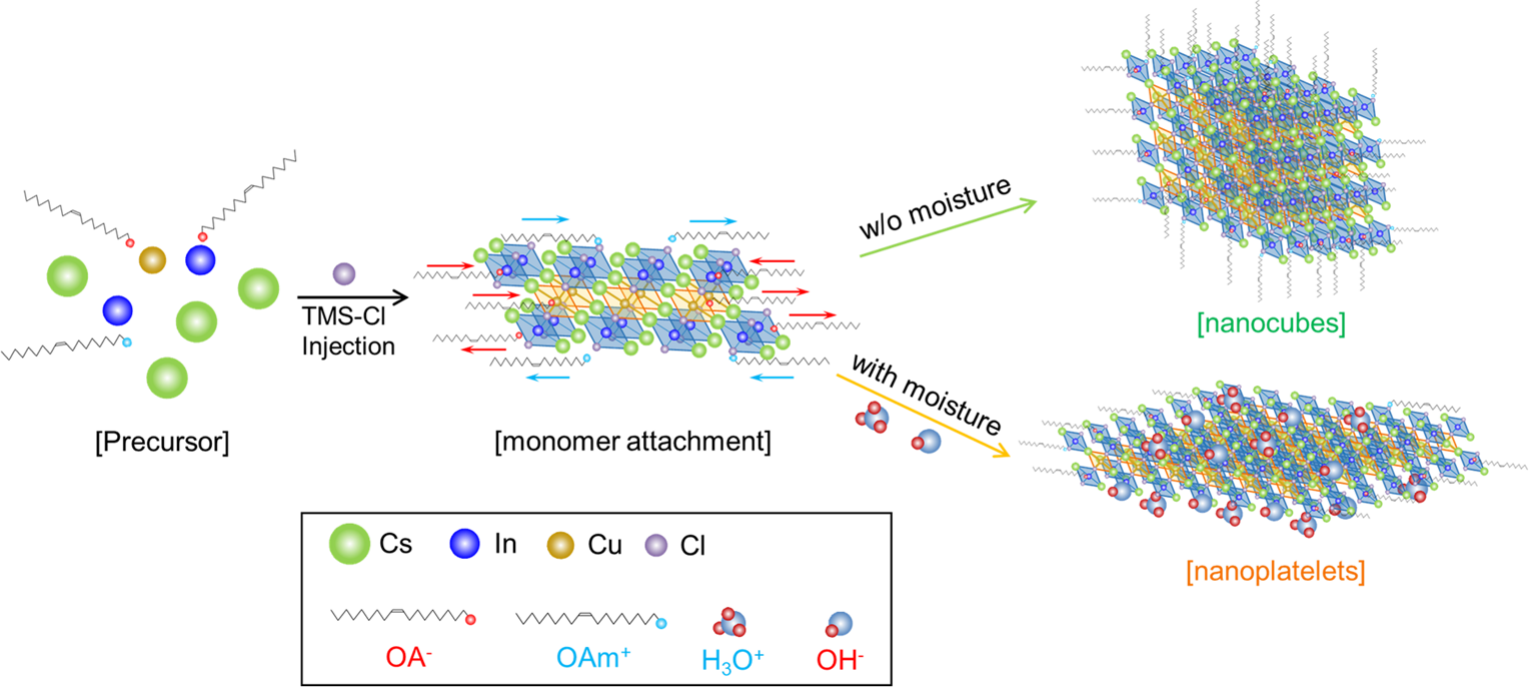
Possible Formation Process of Cs_4_CuIn_2_Cl_12_ NCs Synthesized with and without Moisture in the Precursor

To thoroughly investigate the photophysical properties of Cs_4_CuIn_2_Cl_12_ NCs, we conducted ultrafast transient absorption (TA) measurements for as-synthesized Cs_4_CuIn_2_Cl_12_ NCs. Panels a and b of Figure 3 show the TA spectra of d-Cs_4_CuIn_2_Cl_12_ and w-Cs_4_CuIn_2_Cl_12_ NCs, respectively, excited at 320 nm. A broad positive photoinduced absorption (PIA) was observed in the probe range of 410–650 nm for both samples, providing direct evidence of STEs.^28^ The ultrafast PIA signal growth (<1 ps) indicates a transition from free excitons to STE trapping.^46^ The TA spectra of w-Cs_4_CuIn_2_Cl_12_ NCs display a clear red-shift, particularly after 2 ps, compared to those of d-Cs_4_CuIn_2_Cl_12_ NCs, revealing an enhanced STE effect upon the involvement of water molecules during the NC growth. Figure 3c compares the TA decays for d-Cs_4_CuIn_2_Cl_12_ and w-Cs_4_CuIn_2_Cl_12_ NCs, monitored at 440 and 460 nm, respectively, which can be fitted well with biexponential and triexponential functions, respectively (see the fitting results in Table S4).

**Figure 3:**
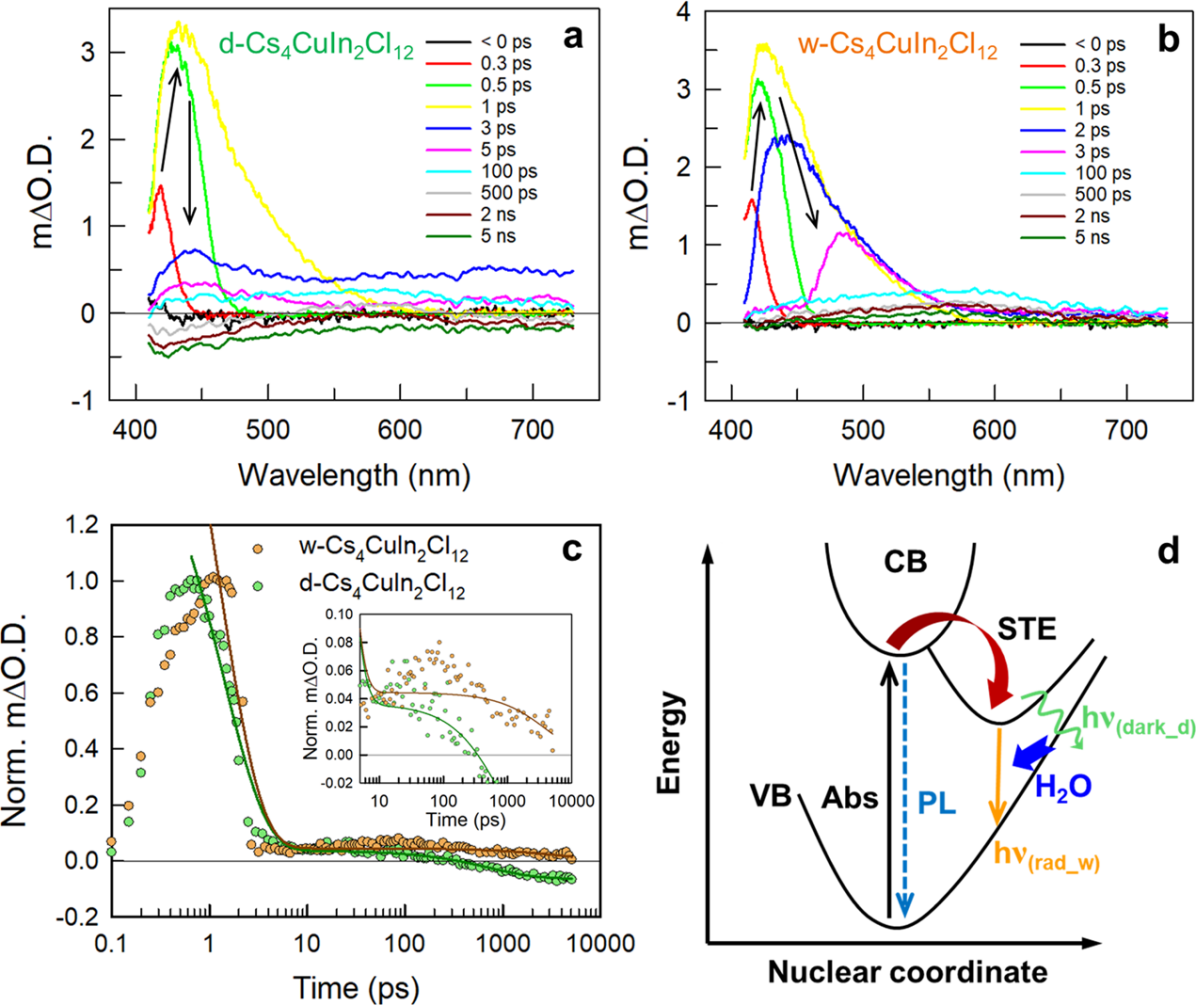
Ultrafast TA spectra of (a) d-Cs_4_CuIn_2_Cl_12_ and (b) w-Cs_4_CuIn_2_Cl_12_ NCs in suspension, excited at 320 nm with an excitation power of 50 μW. The arrows highlight a spectra evolution. (c) TA decays of d-Cs_4_CuIn_2_Cl_12_ and w-Cs_4_CuIn_2_Cl_12_ NCs, monitored at 440 and 460 nm, respectively. Solid lines present the fitting results with a triexponential function:  for w-Cs_4_CuIn_2_Cl_12_ NCs and with a biexponential function:  for d-Cs_4_CuIn_2_Cl_12_ NCs, respectively; ΔOD is the change of optical density. Inset shows the magnified decays in a longer time scale. (d) Configuration coordinate diagram for the STE mechanism of Cs_4_CuIn_2_Cl_12_ NCs with (rad_w) and without (dark_d) moisture in the reaction.

For the case of w-Cs_4_CuIn_2_Cl_12_ NCs, we assign two fast processes comprising an ultrafast component (lifetime of <2 ps) and a medium-fast component (lifetime of 700–800 ps) to nonradiative transitions and a slow component (lifetime of >3 ns) to the radiative recombination of STEs.^47^ Nevertheless, the TA decay for d-Cs_4_CuIn_2_Cl_12_ NCs shows a negligible slow radiative component (third component) with a negative signal (Figure 3c, inset). The 2D w-Cs_4_CuIn_2_Cl_12_ NPLs exhibit an extended TA decay lifetime (τ_AVG_ = 3245.5 ps) by a factor of more than 4 compared to that (τ_AVG_ = 738.2 ps) of the 3D d-Cs_4_CuIn_2_Cl_12_ NCus, indicating that the water molecules induced Jahn–Teller distortion in [CuCl_6_] octahedra can indeed prolong the STE lifetime and result in the radiative transition in the 2D NPLs rather than the dark transition in the 3D NCus with the absence of moisture during the NC formation. This suggests that the water molecules indeed produce a Jahn–Teller distortion in [CuCl_6_] octahedra that involves the contraction of the four horizontal Cu–Cl bonds for w-Cs_4_CuIn_2_Cl_12_ NCs, resulting in a longer STE lifetime and radiative transition compared to the dark transition in d-Cs_4_CuIn_2_Cl_12_ NCs showing weak UV emission (see the STE mechanism of Cs_4_CuIn_2_Cl_12_ NCs in Figure 3d).

In summary, for the first time, we have successfully synthesized lead-free Cs_4_CuIn_2_Cl_12_ layered double perovskite NCs. The critical role of moisture (RH ∼ 40%) during the growth of the NCs has been investigated. It not only led to significantly enhanced PLQYs in the near-UV range, higher structural stability, and morphological transformation from 3D nanocubes to 2D nanoplatelets but also induced a conversion of dark transitions to radiative transitions for STEs in the excited state. Our study provides an effective approach to tune the PL emission in layered double perovskites by tailoring the octahedral distortion in the presence of water molecules. The results presented here will trigger further understanding of PL and photophysical properties for lead-free double perovskites.

## Supporting Information Available

Experimental details, Tauc plots, simulated density of states, simulated structural properties, structural stability, EDS layered images, elemental analysis, ICP-MS data, XPS spectra, low Bragg angle analysis, fitting results of transient absorption decays, and morphological study (PDF)
