# Supplementary material for: Moisture-Assisted near-UV Emission Enhancement of Lead-Free Cs4CuIn2Cl12 Double Perovskite Nanocrystals
Source: Nano Lett. 2021 Dec 23;22(1):311–8. doi: 10.1021/acs.nanolett.1c03822 (PMC8759080; doi:10.1021/acs.nanolett.1c03822)
Supplement: Supplementary file 1 — nl1c03822_si_001.pdf [file nl1c03822_si_001.pdf]

# Supporting Information

# Moisture-Assisted near-UV Emission Enhancement of Lead-free $Cs_4CuIn_2Cl_{12}$ Double Perovskite Nanocrystals

*Maning Liu,*[1,‡] Sri Kasi Matta,[2,‡] Harri Ali-Löytty,[3] Anastasia Matuhina,[1] G. Krishnamurthy Grandhi,[1] Kimmo Lahtonen,[4] Salvy P. Russo,[2] Paola Vivo*[1]*

[1]Hybrid Solar Cells, Faculty of Engineering and Natural Sciences, Tampere University, P.O. Box 541, FI-33014 Tampere University, Finland

[2]Australian Research Council Centre of Excellence in Exciton Science, School of Science, RMIT University, Melbourne, Victoria, 3000, Australia

[3]Surface Science Group, Photonics Laboratory, Tampere University, P.O. Box 692, FI-33014 Tampere University, Finland

[4]Faculty of Engineering and Natural Sciences, Tampere University, P.O. Box 692, FI-33014 Tampere, Finland

*Email: maning.liu@tuni.fi.

*Email: paola.vivo@tuni.fi.

# Table of Contents

**1. Experimental section** ........ 3

*1.1 Chemicals* ........ 3

*1.2 Synthesis of $Cs_4CuIn_2Cl_{12}$ nanocrystals (NCs)* ........ 3

*1.3 Structural simulation* ........ 3

*1.4 Characterization* ........ 4

**2. Results** ........ 6

*2.1 Tauc plots of $Cs_4CuIn_2Cl_{12}$ NCs* ........ 6

*2.2 Simulated density of states (DOS)* ........ 6

*2.3 Structural properties* ........ 6

*2.4 Structural stability* ........ 7

*2.5 Elemental analysis* ........ 7

*2.6 XPS spectra of $Cs_4CuIn_2Cl_{12}$ NCs* ........ 8

*2.7 Low Bragg angle analysis* ........ 8

*2.8 Fitting results of transient absorption dynamic data* ........ 9

*2.9 Morphological study for w-$Cs_4CuIn_2Cl_{12}$ NPLs* ........ 9

**References** ........ 9

## 1. Experimental section

### *1.1 Chemicals*

Cesium acetate (CsOAc, 99.99%), copper (II) acetate ($Cu(OAc)_2$, 99.99%), indium acetate ($In(OAc)_3$, 99.99%), ethyl acetate ( ≥ 99.5%), oleic acid (OA, technical grade, 90%), oleylamine (OAm, technical grade, 70%), 1-octadecene (ODE, technical grade, 90%), chlorotrimethylsilane (TMS-Cl, ≥99%), hexane (≥95%), and toluene were purchased from Sigma Aldrich. All chemicals were used without further purification.

### *1.2 Synthesis of $Cs_4CuIn_2Cl_{12}$ nanocrystals (NCs)*

Colloidal $Cs_4CuIn_2Cl_{12}$ NCs were synthesized based on a modified hot injection method.[1,2] In a typical synthesis, CsOAc (136.2 mg, 0.71 mmol), $Cu(OAc)_2$ (45.4 mg, 0.25 mmol), $In(OAc)_3$ (146.8 mg, 0.50 mmol) were first mixed with oleic acid (OA, 2.5 mL), oleylamine (OAm, 0.66 mL), and octadecene (ODE, 10 mL) in a 25 mL three-neck flask. As a standard procedure to obtain a "dry" precursor, the resulting solution was directly heated to 120 °C under vacuum and degassed for 1 hour to fully eliminate the water and oxygen contents. On the other hand, to create a "wet" precursor, the mixed solution was first degassed at room temperature (RT) for 15 mins to remove the oxygen content while retaining most of the moisture content in the precursor since all operations before degassing were conducted in ambient conditions with an RH of ~40%. Then the wet precursor was further heated to 120 °C in Ar atmosphere for 1 hour to achieve a fully dissolved solution showing a blue color, at which point the same treatment was afterward applied for both dry and wet precursors. The dissolved solution was then heated to 180 °C in Ar atmosphere and 0.5 mL of TMS-Cl was swiftly injected under vigorous stirring. The dry or wet solution quickly turned into a turbid whitish or violet color, respectively, exhibiting the formation of NCs. The reaction was kept at the injection temperature for 1 min before an ice-cold water batch was set under the three-neck flask to quench the further growth of NCs. After cooling to RT, the crude solution was then purified twice with an equal volume of ethyl acetate to remove the unreacted precursors and by-products, followed by centrifugation for 10 min at 4,500 rpm. The resulting precipitate was dispersed in either hexane or toluene for further characterization.

### *1.3 Structural simulation*

The quantum mechanical computations for this study were done using density functional theory (DFT) *ab-initio* first-principles as implemented in Vienna Ab initio

simulation package (VASP) software.[3,4] The structure relaxation was done based on Perdew-Burke-Ernzerhof form (PBE) exchange-correlation functional through generalized gradient approximation (GGA).[5] A Gamma centred k-points mesh[6] of 3×5×3 for $Cs_4CuIn_2Cl_{12}$ bulk material was used in this optimization. The k-points grid was chosen based on the supercell size that was used for optimization. The structure relaxation was done with a convergence criterion for energy and for the force at $10^{-6}$ eV and 0.005eV/Å, respectively. The ionic relaxation was done using a conjugate gradient algorithm. Periodic boundary conditions were applied. The energy cut-off for using the plane-wave basis sets was kept at 450 eV for Projector augmented wave (PAW) method[7] with pseudopotentials. The Grimme's correction scheme[8,9] was used to effectively describe non-covalent bond interactions. The optimized structure was then post-processed using VESTA software[10], and the XRD powder diffraction simulation was done with wavelengths of kα1=1.54059 and kα2=1.54432 and the ratio of kα2: kα1 was kept at 0.5, to match with the experimental conditions. A static run to generate charge density files and wavefunction parameters was done to compute orbital resolved density of states (DOS) for the models. A dense k-point mesh of 11×15×11 is used for DOS.

*1.4 Characterization*

Ultraviolet and visible absorption (UV−vis) spectra were recorded with a dual-beam grating Shimadzu UV-1800 absorption spectrometer. Photoluminescence (PL) spectra and absolute quantum yield (QY) were obtained with an FLS1000 spectrofluorometer (Edinburgh Instruments, UK).

X-ray diffraction (XRD) patterns were measured with Malvern Panalytical Empyrean multipurpose diffractometer with Cu $K_\alpha$ radiation (λ = 1.54059 Å) and a cathode voltage and current of 45 kV and 40 mA, respectively. The samples were scanned over 2θ = 10–60° with a step size of 0.026° and step duration of 17 s per step.

Electron microscopy images were obtained using a transmission electron microscope (TEM) operated at 200 kV (JEM-F200). Energy dispersive X-ray spectroscopy (EDS) was performed using Zeiss UltraPlus FE-SEM electron microscope equipped with Oxford Instruments X-MaxN 80 EDS detector. Samples were drop casted on silicon substrates, and analyzed using 15 kV acceleration voltage. Inductively coupled plasma mass spectroscopy (ICP-MS) measurements were conducted with Thermo Scientific iCAP™ RQ equipment. After purification, NC precipitate samples were dissolved in concentrated $HNO_3$ followed by dilution

and measurement in 2% $HNO_3$ matrix. Ionic standard solutions with a concentration range of 0.001–1000 µg/l for Cs, Cu, and In were prepared in 2% $HNO_3$ using super-pure chemicals (Romil-SpA™) and applied to measure calibration curves. Ultrapure $H_2O$ (18.2 MΩ cm, Merck Milli-Q®) was used for sample dilutions. X-ray photoelectron spectroscopy (XPS) measurements were conducted in an ultrahigh vacuum (UHV) system. The NCs film samples were drop casted on fluorine doped tin oxide (FTO) coated glass slides and let dry in a vacuum desiccator overnight before loading into the UHV system. XPS data were measured using non-monochromatized Al Ka X-rays (hν = 1486.6 eV) generated by a twin anode X-ray source (8025 Twin anode X-Ray source, V. G. Microtech) and a hemispherical electron spectrometer (CLAM4 MCD LNo5, V. G. Microtech). The chemical states of the elements were determined from the XPS spectra by least-squares fitting of asymmetric Gaussian–Lorentzian lineshapes after background subtraction. The analysis was made in CasaXPS software version 2.3.17PR1.1[11] using the Scofield photoionization cross-sections as relative sensitivity factors.[12] The binding energy scale was calibrated according to C 1s (C–C/H) set to 284.8 eV.

Ultrafast transient absorption (TA) measurements were conducted with a standard pump-probe system. The samples were excited by laser pulses at 320 nm (Libra F, Coherent Inc., coupled with Topas C, Light Conversion Ltd.) while a white continuum generator (heavy water) was used to produce the probe beam. The TA responses (time resolution: 150 fs) were measured using an ExciPro TA spectrometer (CDP, Inc.) equipped with a CCD array.

## 2. Results

### *2.1 Tauc plots of $Cs_4CuIn_2Cl_{12}$ NCs*

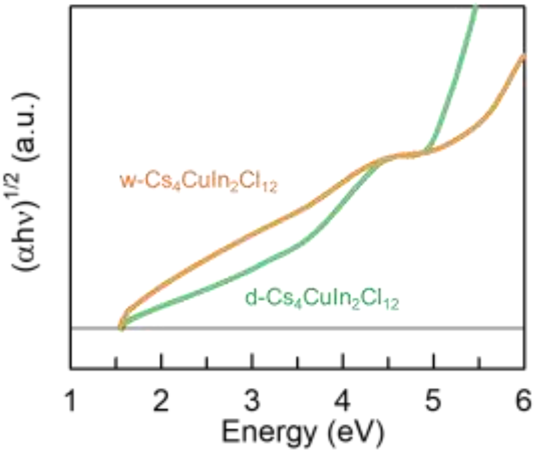


**Figure S1.** Tauc plots of $Cs_4CuIn_2Cl_{12}$ NCs with an indirect transition nature.

### *2.2 Simulated density of states (DOS)*

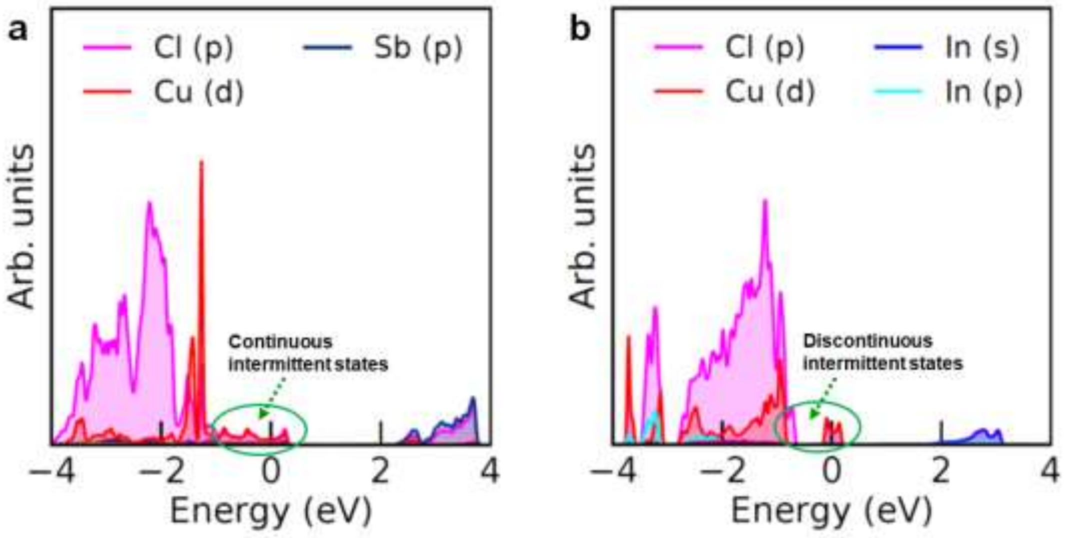


**Figure S2.** Density of States (DOS) of (a) $Cs_4CuSb_2Cl_{12}$ and (b) $Cs_4CuIn_2Cl_{12}$ double perovskites.

In the case of $Cs_4CuSb_2Cl_{12}$, when combining with Cu 3d and 4s orbitals, 5p orbitals of Sb contribute more to the conduction band (CB) energy levels than to the valence band (VB) during the band formation. For $Cs_4CuIn_2Cl_{12}$, the 5p orbitals contribute instead more to the VB rather than to the CB. This explains the high energy difference between the VB and CB energy levels.

### *2.3 Structural properties*

**Table S1.** Computationally optimized crystal structural properties of $Cs_4CuIn_2Cl_{12}$ NCs.

| Space group | Lattice parameters | | | | | | Unit cell volume |
|---|---|---|---|---|---|---|---|
| | *a* | *b* | *c* | *α* | *β* | *γ* | |
| C2 / *m* | 13.18 Å | 7.29 Å | 13.16 Å | 90.0° | 111.8° | 90° | 1174.6 Å$^3$ |

## *2.4 Structural stability*

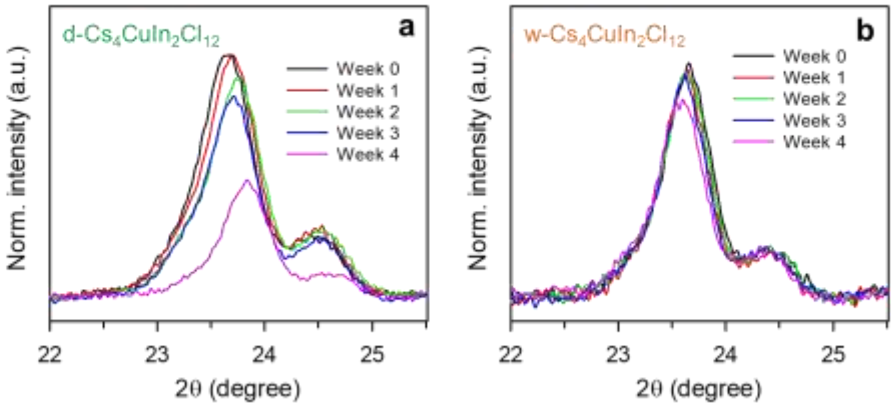


**Figure S3.** Storage time dependent XRD patterns of (a) d-$Cs_4CuIn_2Cl_{12}$ and (b) w-$Cs_4CuIn_2Cl_{12}$ NCs. The NCs films were stored under ambient conditions (RH: ~40%) at RT.

## *2.5 Elemental analysis*

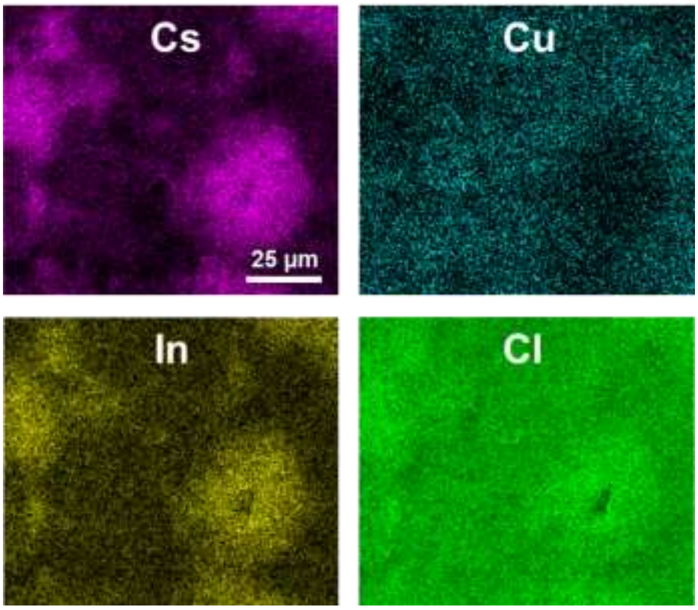


**Figure S4.** Energy-dispersive X-ray spectroscopy (EDS) layered images of Cs, Cu, In, and Cl for w-$Cs_4CuIn_2Cl_{12}$ NCs formed with moisture in the reaction in film state.

**Table S2.** Elemental analysis for $Cs_4CuIn_2Cl_{12}$ NCs. The measured atomic concentration (at%) for each element is normalized with respect to Cs for EDS and ICP-MS, and to Cl for XPS, respectively.

| Analytical method | EDS | | | | ICP-MS | | | XPS | | | | |
|---|---|---|---|---|---|---|---|---|---|---|---|---|
| | Cs | Cu | In | Cl | Cs | Cu | In | Cs | Cu | In | Cl | O |
| d-$Cs_4CuIn_2Cl_{12}$ | 4.0 | 0.7 | 1.9 | 12.0 | 4.0 | 1.1 | 1.9 | 1.2 | 1.2 | 1.8 | 12 | - |
| w-$Cs_4CuIn_2Cl_{12}$ | 4.0 | 0.8 | 1.9 | 11.9 | 4.0 | 1.0 | 2.0 | 0.5 | 1.0 | 1.7 | 12 | 0.8 |

**Table S3.** ICP-MS (inductively coupled plasma mass spectrometry) data for the resultant $Cs_4CuIn_2Cl_{12}$ NCs synthesized with and without moisture in the reaction.

| Samples / µmol $L^{-1}$ | 133Cs (KED) | 63Cu (KED) | 115In (KED) |
|---|---|---|---|
| d-$Cs_4CuIn_2Cl_{12}$ | 0.260 | 0.075 | 0.i129 |
| w-$Cs_4CuIn_2Cl_{12}$ | 0.244 | 0.062 | 0.122 |

## *2.6 XPS spectra of $Cs_4CuIn_2Cl_{12}$ NCs*

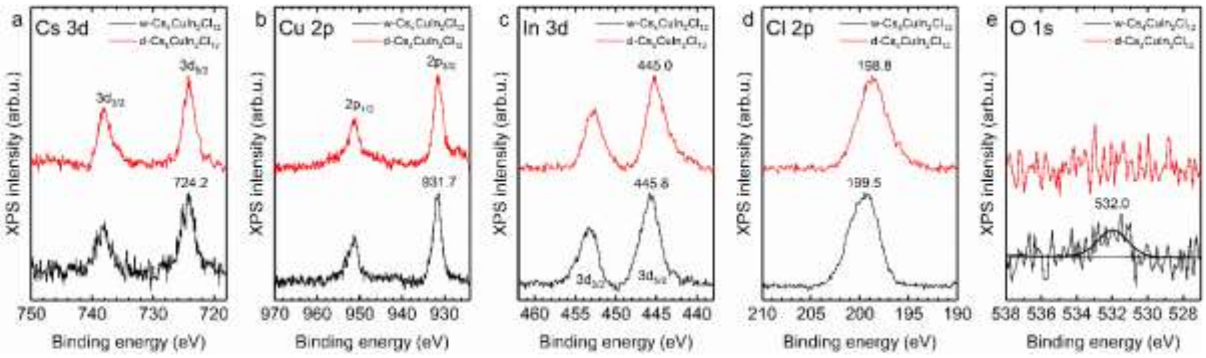


**Figure S5.** XPS spectra of Cs 3d, Cu 2p, In 3d, Cl 2p and O 1s for w-$Cs_4CuIn_2Cl_{12}$ and d-$Cs_4CuIn_2Cl_{12}$ NCs samples in film.

## *2.7 Low Bragg angle analysis*

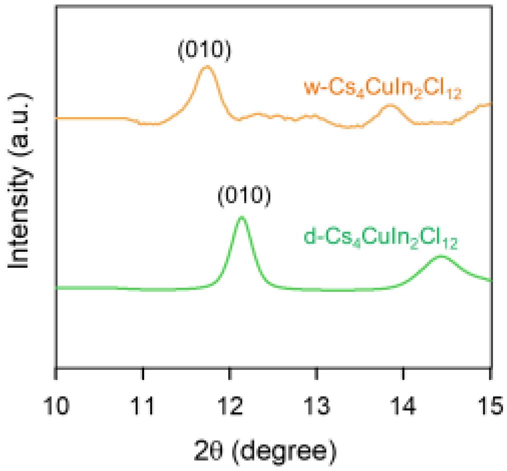


**Figure S6.** XRD patterns of d-$Cs_4CuIn_2Cl_{12}$ and w-$Cs_4CuIn_2Cl_{12}$ in the low-angle (i.e., 10-15°) range.

## 2.8 Fitting results of transient absorption dynamic data

**Table S4.** Fitting results of transient absorption decays for $Cs_4CuIn_2Cl_{12}$ NCs synthesized with and without moisture in the reaction.

| NCs | $A_1$ (%) | $\tau_1$ (ps) | $A_2$ (%) | $\tau_2$ (ps) | $A_3$ (%) | $\tau_3$ (ps) | $^{a)}\tau_{AVG}$ (ps) |
|---|---|---|---|---|---|---|---|
| d-$Cs_4CuIn_2Cl_{12}$ | 94.4 | 1.4 | 5.6 | 761.0 | – | – | 738.2 |
| w-$Cs_4CuIn_2Cl_{12}$ | 92.3 | 1.2 | 4.1 | 782.6 | 3.6 | 3842.7 | 3245.5 |

$^{a)}\tau_{AVG} = \frac{\sum_{i=1}^{n} A_i \tau_i^2}{\sum_{i=1}^{n} A_i \tau_i}$

## 2.9 Morphological study for w-$Cs_4CuIn_2Cl_{12}$ NPLs

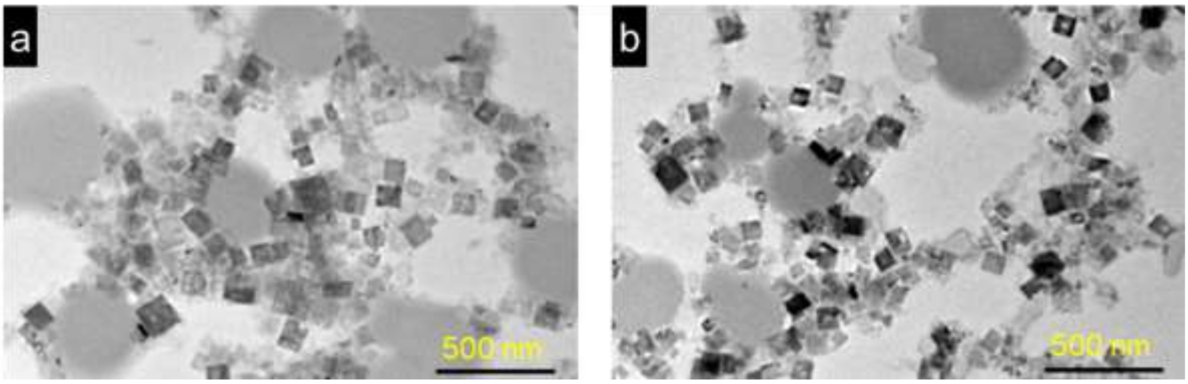


**Figure S7.** TEM images of (a) fresh and (b) aged 2D w-$Cs_4CuIn_2Cl_{12}$ NPLs after 6 days of storage in a nitrogen-filled glove box.

## References


(1) Yang, B.; Hong, F.; Chen, J.; Tang, Y.; Yang, L.; Sang, Y.; Xia, X.; Guo, J.; He, H.; Yang, S.; Deng, W.; Han, K. Colloidal Synthesis and Charge-Carrier Dynamics of $Cs_2AgSb_{1-y}Bi_yX_6$ (X: Br, Cl; $0 \le y \le 1$) Double Perovskite Nanocrystals. *Angew. Chemie - Int. Ed.* **2019**, *58* (8), 2278–2283.

(2) Cai, T.; Shi, W.; Hwang, S.; Kobbekaduwa, K.; Nagaoka, Y.; Yang, H.; Hills-Kimball, K.; Zhu, H.; Wang, J.; Wang, Z.; Liu, Y.; Su, D.; Gao, J.; Chen, O. Lead-Free $Cs_4CuSb_2Cl_{12}$Layered Double Perovskite Nanocrystals. *J. Am. Chem. Soc.* **2020**, *142* (27), 11927–11936.

(3) Kresse, G.; Furthmüller, J. Efficiency of Ab-Initio Total Energy Calculations for Metals and Semiconductors Using a Plane-Wave Basis Set. *Comput. Mater. Sci.* **1996**, *6* (1), 15–50.

(4) Kresse, G.; Furthmüller, J. Efficient Iterative Schemes for Ab Initio Total-Energy Calculations Using a Plane-Wave Basis Set. *Phys. Rev. B - Condens. Matter Mater. Phys.* **1996**, *54* (16), 11169–11186.

(5) Perdew, J. P.; Burke, K.; Ernzerhof, M. Generalized Gradient Approximation Made

Simple. *Phys. Rev. Lett.* **1996**, *77* (18), 3865–3868.

(6) Monkhorst, H. J.; Pack, J. D. Special Points for Brillouin-Zone Integrations. *Phys. Rev. B* **1976**, *13* (12), 5188–5192.

(7) Blöchl, P. E. Projector Augmented-Wave Method. *Phys. Rev. B* **1994**, *50* (24), 17953–17979.

(8) Grimme, S. Semiempirical GGA-Type Density Functional Constructed with a Long-Range Dispersion Correction. *J. Comput. Chem.* **2006**, *27* (15), 1787–1799.

(9) Grimme, S.; Antony, J.; Ehrlich, S.; Krieg, H. A Consistent and Accurate Ab Initio Parametrization of Density Functional Dispersion Correction (DFT-D) for the 94 Elements H-Pu. *J. Chem. Phys.* **2010**, *132* (15), 24103.

(10) Momma, K.; Izumi, F. VESTA 3 for Three-Dimensional Visualization of Crystal, Volumetric and Morphology Data. *J. Appl. Crystallogr.* **2011**, *44*, 1272–1276.

(11) Fairley, N. CasaXPS: Spectrum Processing Software for XPS, AES and SIMS, Version 2.3. 13. *Casa Softw. Ltd* **2006**.

(12) Band, I. M.; Kharitonov, Y. I.; Trzhaskovskaya, M. B. Photoionization Cross Sections and Photoelectron Angular Distributions for X-Ray Line Energies in the Range 0.132-4.509 KeV Targets: $1 \leq Z \leq 100$. *Atomic Data and Nuclear Data Tables*. **1979**, *23* (5), 443–505.
